# A Finite-Element Simulation of Galvanic Coupling Intra-Body Communication Based on the Whole Human Body

**DOI:** 10.3390/s121013567

**Published:** 2012-10-09

**Authors:** Yong Song, Kai Zhang, Qun Hao, Lanxin Hu, Jingwen Wang, Fuzhou Shang

**Affiliations:** School of Opto-Electronics, Beijing Institute of Technology, Beijing 100081, China; E-Mails: 20040491@bit.edu.cn (K.Z.); qhao@bit.edu.cn (Q.H.); hulanxin269@yahoo.com.cn (L.H.); kedawangjingwen@163.com (J.W.); 2120100657@bit.edu.cn (F.S.)

**Keywords:** intra-body communication, finite-element, wireless body area networks, simulation

## Abstract

Simulation based on the finite-element (FE) method plays an important role in the investigation of intra-body communication (IBC). In this paper, a finite-element model of the whole body model used for the IBC simulation is proposed and verified, while the FE simulation of the galvanic coupling IBC with different signal transmission paths has been achieved. Firstly, a novel finite-element method for modeling the whole human body is proposed, and a FE model of the whole human body used for IBC simulation was developed. Secondly, the simulations of the galvanic coupling IBC with the different signal transmission paths were implemented. Finally, the feasibility of the proposed method was verified by using *in vivo* measurements within the frequency range of 10 kHz–5 MHz, whereby some important conclusions were deduced. Our results indicate that the proposed method will offer significant advantages in the investigation of the galvanic coupling intra-body communication.

## 1. Introduction

Intra-body communication (IBC) is a technology that involves using the human body as a transmission medium for electrical signals [1]. Compared with the current short distance wireless communication technologies, such as Bluetooth, Zigbee and so on, IBC has the characteristics of high transmission quality, high security, easy network access and high data rates, *etc.* As a result, IBC technology is proposed as a novel and promising technology for Wireless Body Area Networks (WBANs) [1,2], biomedical monitoring [3,4] and supplying power for implants [4], *etc.* On the other hand, galvanic coupling IBC is an important approach to achieve the signal transmission within the human body [5,6]. In this approach, signal transmission is achieved by coupling signal currents galvanically into the human body. The electrical signal is applied over a pair of transmitting electrodes and thereby establishes an electrical field, and then it is received by the receiver over a pair of receiving electrodes. Compared with the other types of IBC, the signal transmission quality of the galvanic coupling IBC is not influenced by the individual's surroundings [6,7]. Meanwhile, miniaturized electrodes can also be used in this approach, therefore, galvanic coupling IBC is presented as a promising approach for data communication within the human body.

To guarantee the safety of the human body, many IBC experiments were carried out by using simulation methods. Therefore, software simulation serves as an important method in the investigation of intra-body communication. The software simulation of IBC can be implemented by using the transfer function method [8,9], in which the transfer function of IBC is developed first for describing the mathematical relation of the different parts in the IBC system, then the IBC simulation can be achieved by using the transfer functions. On the other hand, the finite-element method was also chosen in the software simulation of IBC [10,11], in which the finite-element model of the human body parts was developed first, then the IBC simulation was implemented using the developed model. Using this method, the electromagnetic field distribution within the human body can be achieved. Moreover, it's easy to determine the positions of the electrodes attached on the actual human body in a FE model. As a result, the finite-element method is considered as an important one in the investigation of the intra-body communication [10–12]. On the other hand, the FE simulation of the galvanic coupling IBC with different signal transmission paths is important to investigate the characteristics of the human body channels. In the IBC application, signals may transmit from electrical devices located on the arms (such as an electronic watch) to electrical devices located on the torso (cell phone, MP4, *etc.*) or earphones located on the head. Sometimes, signals may transmit from IBC shoes to the other parts of the human body (torso, arms, *etc.*). As a result, the development of the FE model of the whole human body and the investigation of the potential distribution corresponding to different signal transmission paths will help to disclose the signal transmission characteristics of the human body. Unfortunately, due to the complex structure of the human body, it has proved difficult to develop a FE model of the whole human body. For instance, although a precise geometry model of the human body can be achieved by using Magnetic Resonance Imaging (MRI) or Computerized Tomography (CT) technology, the modeling of the whole human body based on these technologies will be a complicated work and result in huge computational cost. Therefore, only the FE model of upper arm has been developed [10–12] by Wegmueller, *et al.*, who first developed an upper arm model and then achieved the simulation of the galvanic coupling IBC [10].

In this paper, a finite-element model of the whole body model used for the simulation of the galvanic coupling IBC is firstly proposed and verified, while the FE simulation of the galvanic coupling IBC with the different signal transmission paths has been achieved. First, we discuss the theoretical foundation of the finite-element method for modeling human body. Secondly, the modeling of the whole human body and the corresponding electromagnetic parameters are described, and then the simulation of the galvanic coupling IBC with the different signal transmission paths are implemented and the corresponding potential distributions are discussed in detail. Finally, the feasibility of the proposed model is verified by using *in vivo* measurements, whereby some important conclusions are deduced.

The rest of the paper is organized as follows: Section 2 focuses on the geometry modeling and the corresponding parameters of the whole human body. The galvanic coupling IBC simulation conditions and the results discussion are presented in Section 3. Section 4 mainly verifies the feasibility of the proposed model by using *in vivo* measurements. Section 5 concludes the paper.

## 2. The Modeling of the Whole Human Body

### 2.1. Theoretical Foundation

The coupling between electromagnetic signals and human body can be explained by the Maxwell equations and the boundary conditions. According to [13], the inductive effects and the wave propagation can be neglected in biological tissues. Therefore, the electric and magnetic fields are decoupled. As a result, the Maxwell equations can be described as:

(1) $$\{\begin{array}{l} \nabla \times \bar{H}=\bar{J}+\partial D/\partial t\\ \nabla \times \bar{E}\approx 0\\ \nabla \cdot \bar{B}=0\\ \nabla \cdot \bar{D}=\rho \end{array}$$

In the galvanic coupling IBC application, due to the fact that electronic signal is transmitted and received as electronic form, as a result, only the electric field is of interest [3,10]. Therefore, the Maxwell equations can be simplified using the continuity equation and the constitutive relations. Due to the fact that *J̅* = *σE̅* and *D̅* = *εE̅*, then the quasistatic electric field of the intra-body communication can be described as follows [10]:

(2) $$-\nabla \cdot (ɛ\nabla V)+\frac{i}{\omega }\nabla \cdot (\sigma \nabla V)=0$$

Based on the equations mentioned above, the electronic signal transmission within the human body can be simulated by using the finite-element method, which is applied to solve the quasi-static volume conducting boundary problem by the numerical solution of the partial differential equations.

### 2.2. The Geometry Modeling

**Figure 1.**
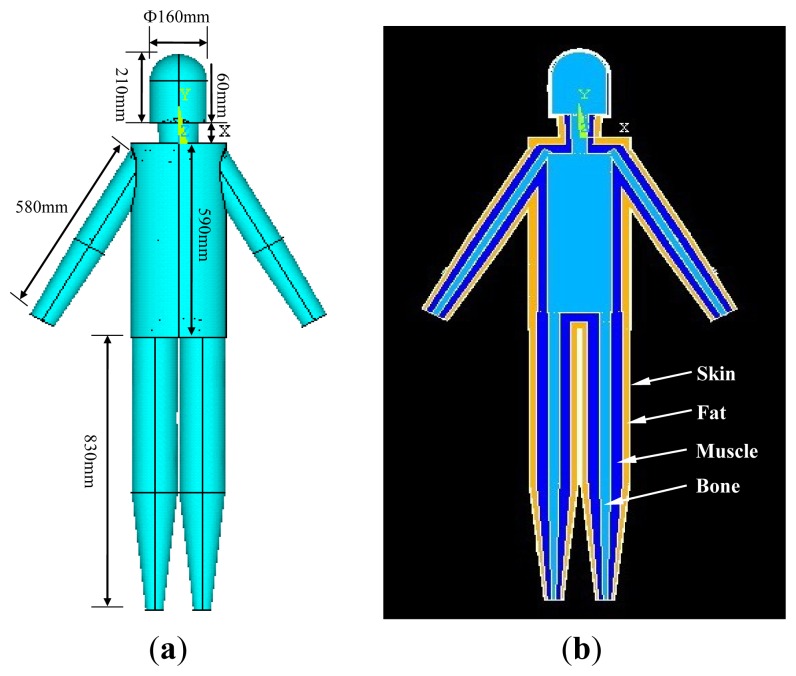
The 3-D view (**a**) and the internal structure (**b**) of the whole body model.

A human body part (such as the upper arm) can be abstracted as a concentric cylinder with multiple layers in the simulation of the interaction between the electromagnetic signal and the human body [10,14]. Similarly, the human body can also be abstracted as a model consisting of the cylinders representing different body parts. In our geometry modeling, the developed model of the whole human body consists of head, neck, torso, arm and leg, as shown in Figure 1(a). Firstly, according to the geometry of the male adult [15], the models representing the different body parts were developed. Generally, the geometry of the human parts can be divided into five layers, which include skin, fat, muscle, cortical bone and bone marrow [10]. However, if we develop a whole human body model with these five layers, it will entail a large computational cost and thereby result in comparatively long simulation times. In our investigation, each IBC simulation using a personal computer (CPU: Dual-core 1.8 GHz, Memory: 2 GB DDR, Hard disk: 160 GB) might take more than 12 h. On the other hand, the conductive path of the IBC is mainly formed by the surface layers, which includes skin, fat [12], while a more detailed geometry with complex layer structures would not significantly improve the results [10]. Therefore, to decrease the computational cost, all the body parts of our FE model were divided into four layers, which include, from outside to inside, skin, fat, muscle and bone, as shown in Figure 1(b).

**Figure 2.**
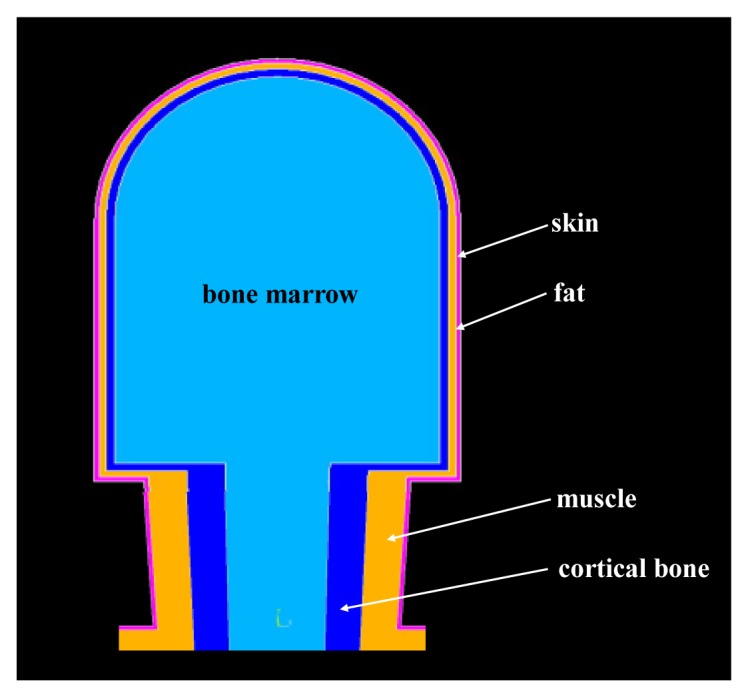
The modeling of head and neck.

**Figure 3.**
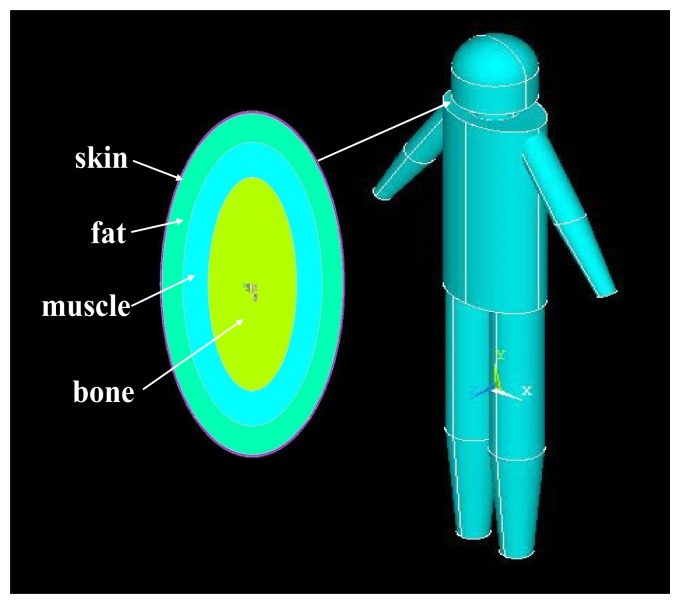
The torso of the whole body model.

1. *The Modeling of Head and Neck*: In our modeling, the head was modeled geometrically as a semi-sphere and a concentric cylinder, which have the same radius of 80 mm, as shown in Figure 2. Due to the fact that brain tissue is encapsulated in the skull cap, the skull cap as well as the brain tissue were treated as the same media (bone layer) in our modeling. Therefore, the head of the proposed model includes, from outside to inside, skin, fat, muscle and bone. Additionally, according to [15], the thicknesses of the different layers were set as 2 mm (skin), 3 mm (fat), 4 mm (muscle) and 71 mm (bone; radius) respectively. As shown in Figure 2, the neck of the human body was abstracted as a cone, in which the major diameter surface was connected with the head while the minor diameter surface was connected with the torso. Similarly, the neck model was also modeled by four layers, while the thicknesses of the neck were set as 2 mm (skin), 17 mm (fat), 17 mm (muscle) and 23 mm (bone; radius) from outside to inside.
2. *The Modeling of Torso*: According to the geometry of the actual torso of human body, the torso of the developed model was abstracted as an elliptic cylinder, which has a long-axis length of 265 mm, a short-axis length of 195 mm and a height of 590 mm, as shown in Figure 3. Similarly, the elliptic cylinder was also modeled by four layers (skin, fat, muscle and bone), while the thickness of these layers were set as 1.7 mm (skin), 22 mm (fat) and 27 mm (muscle), respectively. Moreover, the long-axis length and the short-axis length of the bone layer were set as 154 mm and 84 mm, respectively.
3. *The Modeling of Arm and Leg:* As shown in Figures 1 and 3, both the arms and the legs consist of a cylinder and a cone in the developed model, in which the upper arms were modeled by the cylinders with a length of 32 mm, while the lower arms were modeled by the cones with a length of 26 mm. Moreover, the radius of the lower arms was decreased from 96.7 mm (the major diameter) to 78.3 mm (the minor diameter). Similarly, the thighs of the model were modeled by cylinders with a length of 47 mm, while the shanks were modeled by the cones with a length of 36 mm. Additionally, the radii of the shanks were decreased from 79 mm (the major diameter) to 30 mm (the minor diameter). On the other hand, both the arms and the legs were also modeled by four layers, in which the thickness of the upper arms are 1.7 mm (skin), 12 mm (fat), 23 mm (muscle) and 60 mm (bone, radius), while the thickness of the thighs are 2 mm (skin), 17 mm (fat), 46 mm (muscle) and 14 mm (bone; radius).
4. *The Connection of the Body Part Models:* Finally, the models of the human parts (head, torso, arms and legs, *etc.*) were connected with each other, thereby forming the finite-element model of the whole human body. Generally, the connection of the different FE models can be achieved by using Boolean operations. However, due to the big size and the complexity of the whole human body, this process will generate huge unit numbers and prolong the simulation time. In our modeling, all the nodes within a certain range in the contact surface of the two FE models were combined into one node. As a result, the process time of mesh generation was limited in an acceptable range (30 min).

### 2.3. The Electromagnetic Parameters

The electromagnetic parameters are essential to achieve the IBC simulation based on the finite-element method. The electromagnetic characteristics of human tissues (such as skin, fat, muscle and bone) can be described by their conductivity and relative permittivity. On the other hand, the conductivity and relative permittivity of the different tissues have been investigated by Gabriely *et al.* [16]; some of their results corresponding to the different frequencies are shown in Table 1, in which *σ* represents the conductivity and *ε_r_* represents the relative permittivity. According to the electromagnetic parameters measured in the previous works [16], the conductivities and relative permittivities used in the developed model can be determined.

## 3. The Simulations of Galvanic Coupling IBC

### 3.1. Simulation Conditions

**Figure 4.**
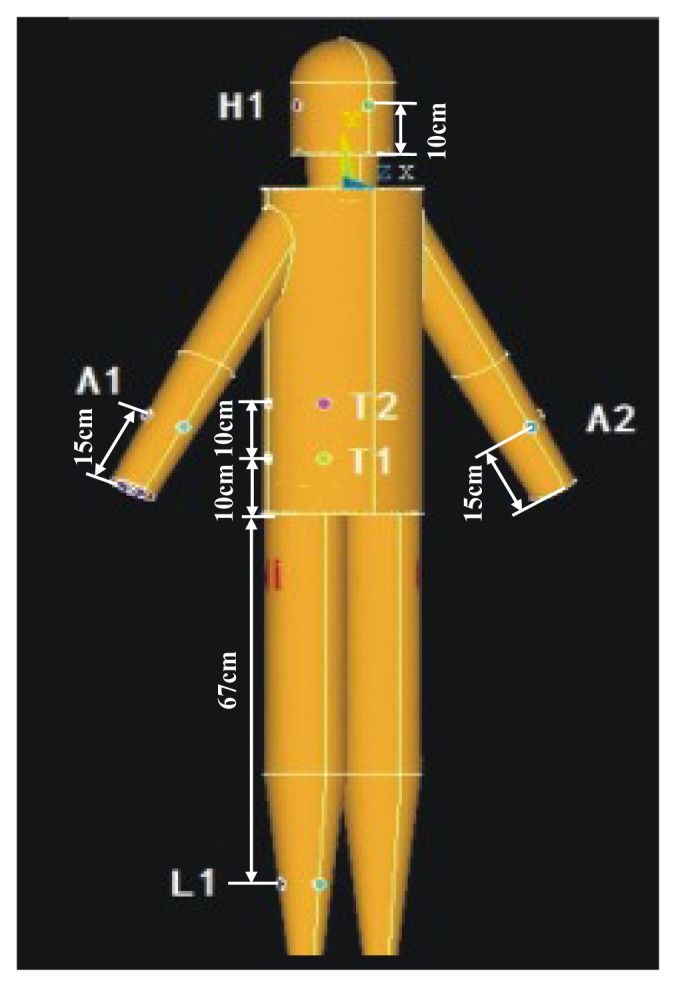
The electrode distribution over the whole body model.

The simulations of the galvanic coupling IBC with different signal transmission paths were carried out using the proposed FE model. The simulations were produced with the electromagnetic analysis package (EMAG) of ANSYS, while the mesh sizes were set between 400,000 and 450,000 elements. To isolate the human body model against air, the normal component of the electric field corresponding to the boundary between the skin and the air was set to zero [10]. Additionally, all the galvanic coupling electrodes were configured as the circular copper electrodes with the radius of 10 mm, while the corresponding conductivity (*σ*) and the relative permittivity (*ε_r_*) were set as 5.99E7 and 1 [10], respectively. Figure 4 shows the particular locations of the electrodes in the simulation, in which the electrodes were distributed on the arm (A1, A2), head (H1), torso (T1, T2) and legs (L1). According to the geometry of the different body parts, the two electrodes of the transmitter or the receiver were separated by 5 cm (arm), 10 cm (head), 8 cm (torso) and 6 cm (leg). On the other hand, a signal of current-controlled harmonic waveform with 1mA amplitude was coupled into the model through the transmitter electrodes, while 10 frequencies (10, 20, 50, 100, 200, 500 kHz, 1, 2, 3 and 5 MHz) were chosen as the signal frequencies used in the simulation.

### 3.2. Simulation Results

**Figure 5.**
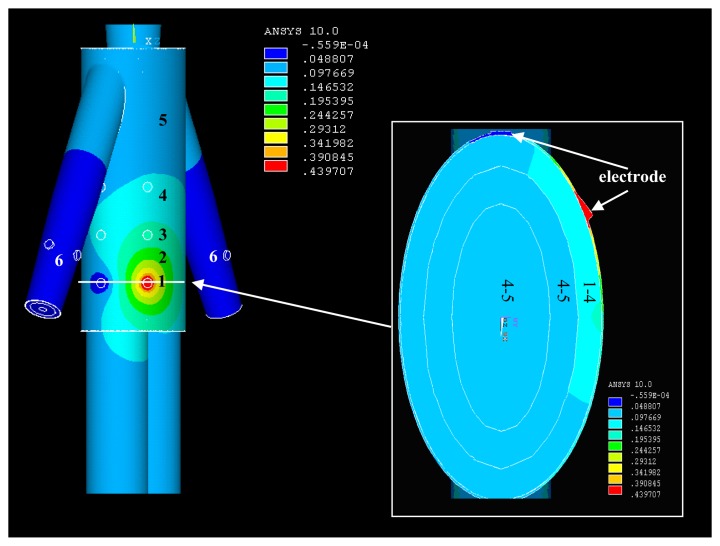
The potential distribution when signal transmits from T1.

**Figure 6.**
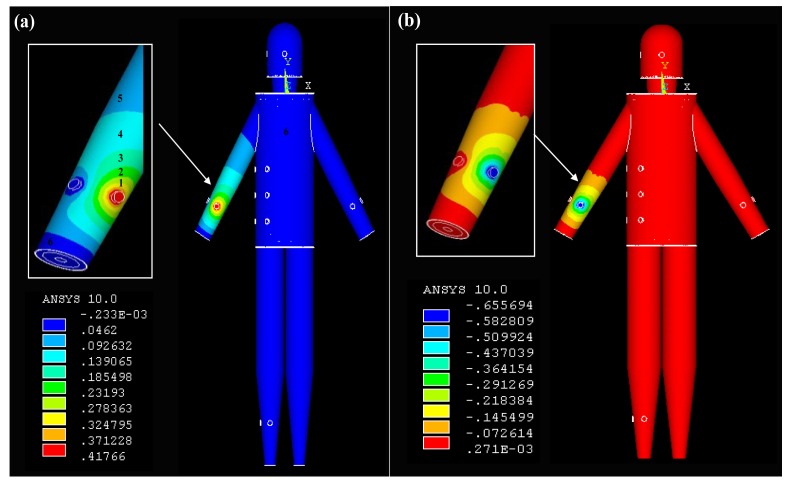
The real part (**a**) and the imaginary part (**b**) of the potential distribution when a signal transmits from the arm.

**Figure 7.**
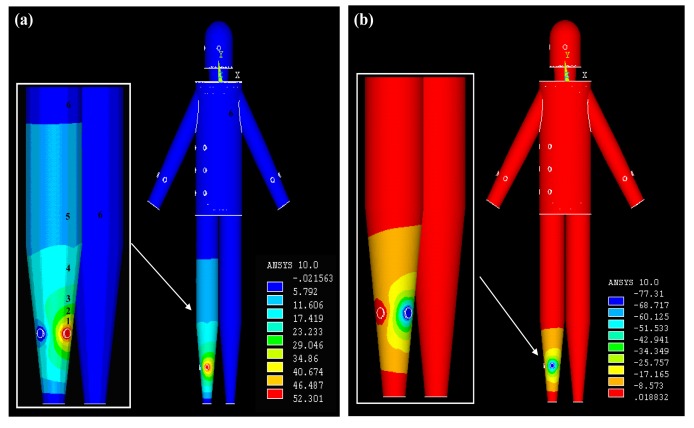
The real part (**a**) and the imaginary part (**b**) of the potential distribution when signal transmits from the leg.

1. *Signal transmits from the torso*: Figure 5 represents the potential distribution of the simulation results (real part) when the signal was coupled into the model at T1, which includes the potential distribution of the torso surface and that of the horizontal cross-section corresponding to T1 within the torso model. It can be observed from Figure 5 that the relatively higher potentials mainly focus on the surroundings of the transmitting electrode injecting current signal into the torso, while the position of T1 has the maximum potential of 0.44 V. Meanwhile, with the increasing of the signal transmission distance, the potential values decrease almost in all the directions. For instance, the potential values decrease from 0.39 V to 0.14 V when the signal transmission distance is increased from Section 1 to Section 3. On the other hand, due to the fact the potential of the other electrode was set to zero, the surrounding of this electrode has an approximately zero potential, as shown in Figure 5. Additionally, the potential values of Section 5 corresponding to some parts of the torso, leg and torso are within 0.10 V–0.05 V, while the maximum potential values of Section 6 corresponding to the lower arms is only 0.05 V.
  We can also find from Figure 5 that the highest potential level (0.44 V–0.24 V) is mainly focused on the skin layer along the long-axis direction of the torso. Meanwhile, the secondary potential level (0.24 V–0.15 V) mainly locates in the fat layer. Moreover, the potential values of the other layers (muscle, bone) within the torso are between 0.05 V and 0.15 V. The results mentioned above indicate that the relatively higher potentials mainly focus the surface layers (skin, fat) in the galvanic coupling IBC, which coincides with the previous results [10].
2. *Signal transmits from the arm:* The potential distribution when the IBC transmitting electrodes were attached on the arm (A1) of the whole body model is shown in Figure 6. It can be found from Figure 6(a) that the potential distribution (real part) of the arm is similar to that of the torso shown in Figure 5. The section with the relatively higher potentials (0.42 V−0.27 V) is mainly focused on the position of the transmitting electrode injecting current signal into the arm. Meanwhile, the potential values decrease from 0.27 V to 0.05 V when the signal transmission distance is increased from Section 1 to Section 5 along the arm axis. On the other hand, Section 6, corresponding to the two terminals of the right arm, the left arm, head, torso and legs has the relatively lowest potential level, which has the maximum values of 0.05 V. Additionally, the similar potential distribution can also be found in Figure 6(b), which shows the imaginary part of the potential distribution when signal transmits from the arm.
3. *Signal transmits from the leg*: Figure 7 is the potential distribution of the simulation results when the IBC transmitting electrodes were attached on the right leg (L1). As shown in Figure 7(a), the right leg also has a similar potential distribution as the right arm shown in Figure 6(a). The position of the transmitting electrode attached on the right leg has the relatively higher potentials (0.52 V−0.29 V). Meanwhile, the potential values decrease from 0.29 V to 0.06 V when the signal transmission distance is increased from Section 1 to Section 5 along the axis of the right leg. Moreover, Section 6, corresponding to some parts of the right leg, left leg, torso, arm and head has the relatively lowest potential level. On the other hand, Figure 7(b) shows the imaginary part of the potential distribution. Although the area with comparatively high potential of Figure 7(b) is bigger than that of Figure 7(a), the similar potential distribution can also be found in the two figures.

## 4. The Measurement Experiments

### 4.1. Measurement Methods

**Figure 8.**
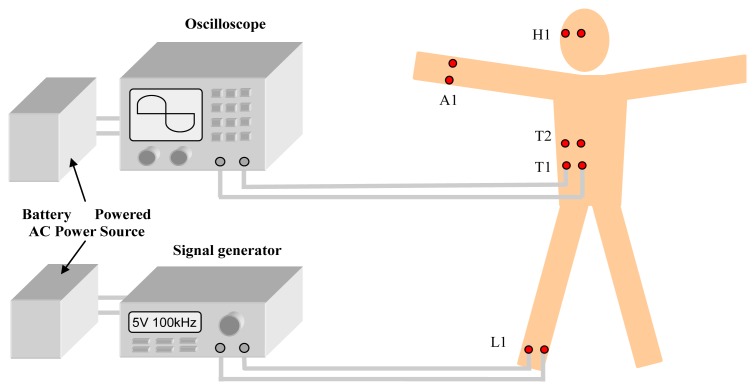
The measurement setup.

To verify the feasibility of the proposed FE model, *in vivo* measurements of the galvanic coupling IBC with the different signal transmission paths were carried out. The measurement setup, as shown in Figure 8, comrised a signal generator, a digital oscilloscope, and the galvanic coupling electrodes, in which the signal generator (SG1040, made by Jiangsu Right Electronic Equipment Company, Ltd., Huai'an, China) was used for simulating IBC transmitter, a digital oscilloscope (Agilent 54641A) was used for simulating the IBC receiver. Additionally, to simulate the application of galvanic coupling IBC in WBANs [2], both the signal generator and the digital oscilloscope were powered by a battery powered AC power source. Meanwhile, a volunteer (male, 24 years old) who has a similar geometry as the proposed FE model of the whole human body was chosen as the subject.

### 4.2. Measurement Results

In the investigation of IBC, signal attenuation can be determined by using 20·log_10_(*U_r_*/*U_t_*) [10], in which *U_r_* and *U_t_* are the input voltage and the receiving voltage of IBC path, respectively. In our calculation of the signal attenuations corresponding to the *in vivo* measurements, *U_r_* is the measured value of the differential voltage between the two receiving electrodes, while *U_t_* is the output voltage of the signal generator. Meanwhile, in order to calculate the signal attenuations corresponding to the IBC simulations, *U_r_* was represented as the potential difference between the two receiving electrodes, while *U_t_* was represented as the potential difference between the two transmitting electrodes.

**Figure 9.**
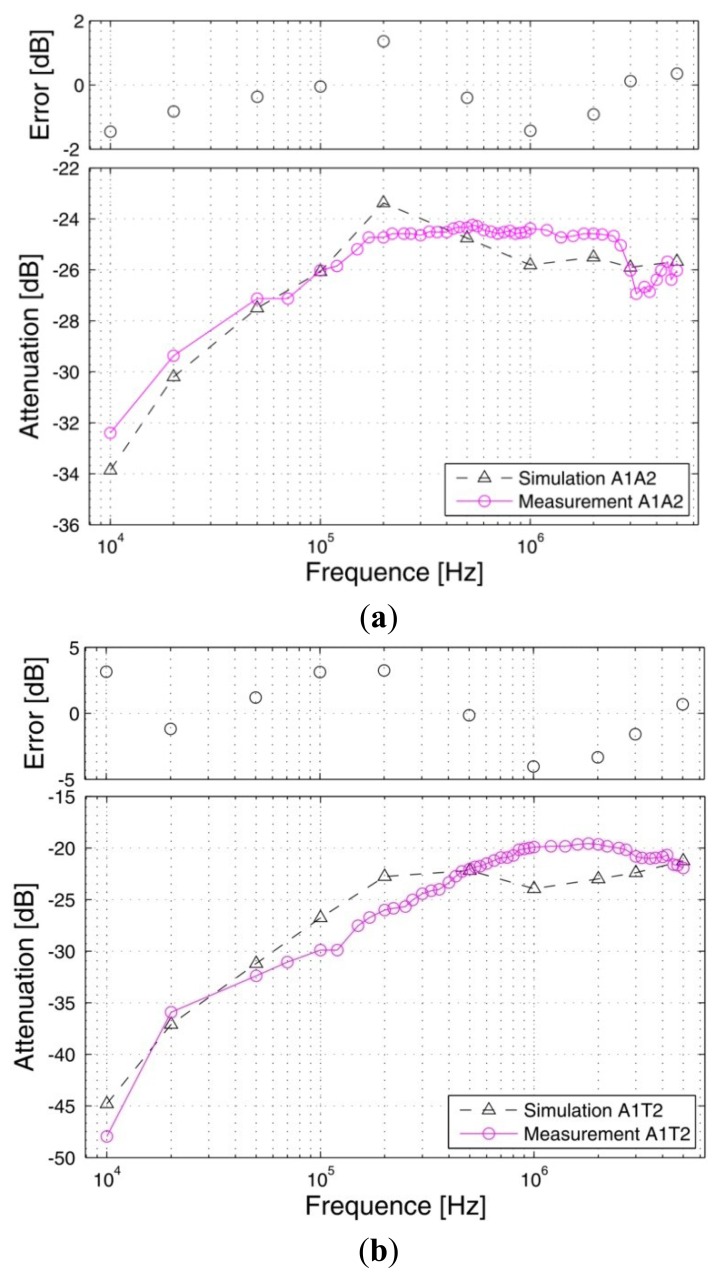

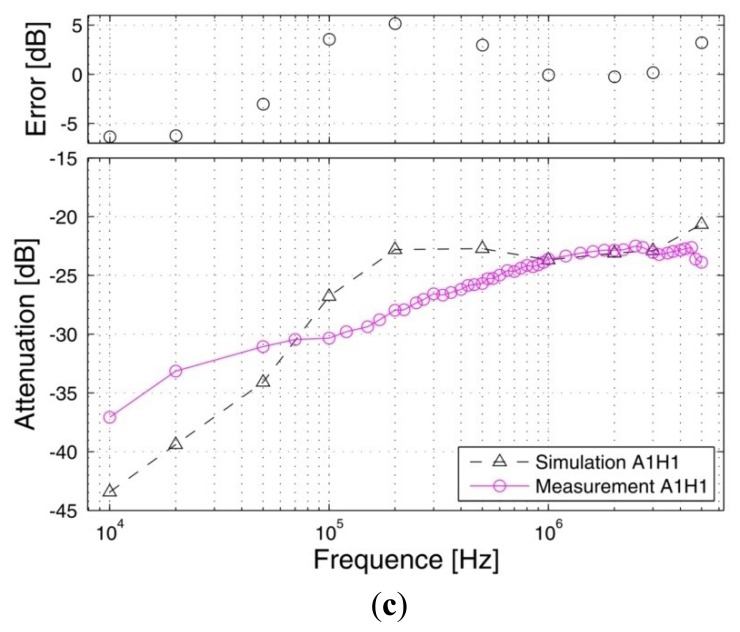
The simulation results and the measurement results of the A1A2 path (**a**), the A1T2 path (**b**) and the A1H1 path (**c**).

**Figure 10.**
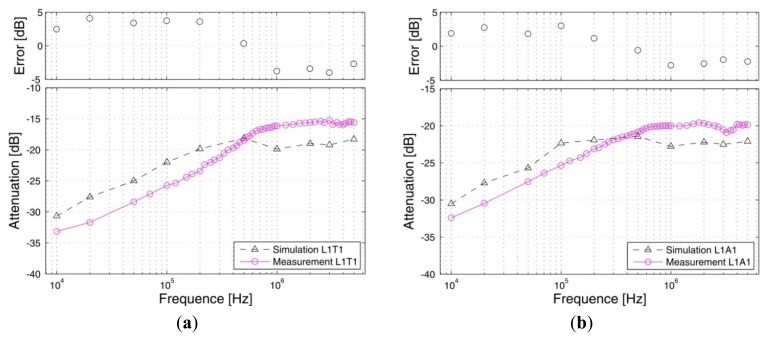
The simulation results and the measurement results of the LIT1 path (**a**) and the L1A1 path (**b**).

**Figure 11.**
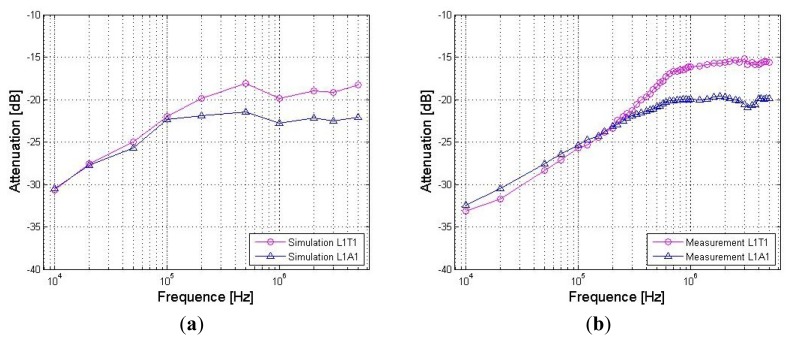
The influences of signal transmission distance on the simulation results (**a**) and the measurement results (**b**).

1. *Signal transmits from the arm*: Figure 9 shows the signal attenuations of the *in vivo* measurements and the simulation results corresponding to the paths starting from the right arm (A1), in which Figure 9(a) shows the results of the path from the right arm to the left arm (A1A2), Figure 9(b) shows the results of the arm-torso path (A1T2) and Figure 9(c) shows the results of the arm-head path (A1H1). The signal transmission distances of the three paths are 116.50 cm (A1A2), 74.00cm (A1T2) and 84.75 cm (A1H1), respectively. Meanwhile, the corresponding errors between the measurement and the simulation are also provided in the figures. It can be seen from Figure 9(a) that the simulation results based on the proposed FE model basically agree with the corresponding *in vivo* measurement results within the 10 kHz–5 MHz frequency range. Both the simulation results and the measurement results decrease gradually as the signal frequency increases from 10 kHz to 200 kHz. For instance, the absolute attenuation value of the simulation result decrease from 33.86 dB to 23.37 dB within the range of 10 kHz to 200 kHz, while the corresponding value of the measurement also decrease from 32.40 dB to 24.73 dB. Meanwhile, both of the two curves keep a slight variation as the signal frequency increase from 200 kHz to 5 MHz. Additionally, the errors between the simulation and the measurement are limited in an acceptable range, while the maximum absolute value of the errors is only 1.46 dB.
  Similarly, we can find from Figure 9(b,c) that the simulation curves and the *in vivo* measurement curves corresponding to the A1T2 and A1H1 paths also have similar outlines, while the absolute values of the errors between the simulation and the measurement are limited to 4.03 dB (AlT2) and 6.36 dB (AlH1), respectively, which indicate that the simulation results corresponding to the two paths basically agree with the corresponding measurement results. Moreover, compared with the A1A2 path, the errors between the simulation and the measurement show a slight increase, which is approximately equal to the corresponding errors of the previous results [10].
2. *Signal transmits from the leg*: Figure 10 shows the signal attenuations of the simulation and measurement results corresponding to the paths starting from the right leg (L1), in which Figure 10(a) shows the two results of the leg-torso path (L1T1) and Figure 10(b) shows that of the leg-arm path (L1A1). It can be seen from Figure 10(a) that the simulation results of the leg-torso path basically agrees with the corresponding measurement results within the 10 kHz–5 MHz frequency range. According to Figure 10(a), both the simulation results and the *in vivo* measurement results decrease as the signal frequency increases from 10 kHz to 500 kHz, while displaying a slight variation as the signal frequency increased from 500 kHz to 5 MHz. For instance, the absolute attenuation value of the simulation result decreases from 30.67 dB to 18.14 dB within the range of 10 kHz–500 kHz, while the corresponding measurement value also decreases from 33.15 dB to 18.49 dB. Meanwhile, the maximum variation range of the simulation result within 500 kHz–5 MHz is 1.75 dB, while that of the measurement results is 3.28 dB. Additionally, the errors between the simulation and the measurement with respect to the frequency range of 10 kHz–5 MHz are limited to 4.10 dB. Similarly, we also can find from Figure 10(b) that the simulation curve and the measurement curve have similar outlines, while the absolute values of the errors between the simulation and the measurement are limited to 3.01 dB, which indicates that the simulation results corresponding to the L1A1 path also basically agree with the corresponding measurement results.
  On the other hand, due to the fact that both the L1T1 path and the L1A1 path consist of leg and torso, as shown in Figures 4 and 7, the influence of the signal transmission distance can be compared by using the results with respect to the two paths, which have signal transmission distances of 77 cm (L1T1) and 178 cm (L1A1), respectively. According to Figure 11, which shows the influences of signal transmission distance on the simulation results and the measurement results, some conclusions may be deduced as follows: (1) the signal transmission distance has relatively less influence on the signal attenuation of the galvanic coupling IBC within the frequency range of 10 kHz–100 kHz. For instance, as the signal frequency increases from 10 kHz to 100 kHz, the measurement result of the L1T1 path decreases from 33.15 dB to 25.75 dB, while that of the L1A1 path also decreases from 32.40 dB to 25.35 dB. Similarly, the simulation result of the L1T1 path decreases from 30.67 dB to 22.01 dB, while that of the L1A1 path also decreases from 30.50 dB to 22.34 dB; (2) with the increasing signal transmission distance, the difference between the signalattenuations of the simulation results with respect to the two paths increases gradually within 100 kHz–500 kHz. Meanwhile, similar phenomenon can also be found in the corresponding measurement results, even though the difference between them is comparatively small within the 100 kHz–200 kHz range; (3) the signal transmission distance has a relatively high influence on the signal attenuation within the 500 kHz–5MHz frequency range. For instance, the average increase of the measurement results within the 500 kHz–5 MHz range is 3.92 dB, while the corresponding value of the simulation results is 3.3 dB. Accordingly, the distance sensitivity of the measurement results and the simulation results are 0.039 dB/cm and 0.033 dB/cm, respectively, which indicates that the proposed FE model has similar sensitivity to the signal transmission distance as the actual human body.

## 5. Conclusions

The development of a FE model of the whole human body and the investigation of the potential distribution corresponding to different signal transmission paths will help to clearly disclose the signal transmission characteristics of the human body. In order to meet this requirement, a finite-element method for modeling the whole human body is proposed in this paper, while both the simulations of the galvanic coupling IBC based on the whole human body and the corresponding *in vivo* measurements have been carried out, and some important conclusions can be drawn as follows: (1) in the galvanic coupling intra-body communication, the relatively higher potentials are mainly focused at the position of the transmitting electrode injecting current signal into the human body, while the potential decreases along the signal transmission path within the body model; (2) according to the simulation results, the relatively higher potential level is mainly focused on the skin layer and the fat layer, while the muscle layer has the relatively lower potential, which is in aggremment with Wegmueller's conclusion [10]; (3) the simulation results based on the proposed FE model basically agree with the corresponding *in vivo* measurement results within the 10 kHz–5 MHz frequency range; (4) the signal transmission distance has relatively less influence on the signal attenuation within the 10 kHz–100 kHz range, while it begins to influence the signal attenuation within the 100 kHz–500 kHz range and has a relatively high influence on the signal attenuation within the 500 kHz–5 MHz range. Additionally, the distance sensitivity of the proposed FE model is similar to that of the actual human body. The proposed finite-element model of the whole human body has been used for the simulations of the signal transmission among the sensors attached on the different parts of the human body. Our next work will focus on the simulations of the signal transmission within implant sensors.

## Figures and Tables

**Table 1.** The conductivities and relative permittivities.

	**Frequency (kHz)**	**Skin**	**Fat**	**Muscle**	**Bone**
*σ*	10	4.0E−03	3.0E−02	3.5E−01	2.0E−02
	20	7.0E−03	3.0E−02	3.5E−01	2.0E−02
	50	1.2E−02	2.9E−02	3.7E−01	2.0E−02
	100	4.0E−02	2.8E−02	3.9E−01	2.0E−02
	200	9.0E−02	3.5E−02	4.4E−01	2.2E−02
	500	1.3E−01	4.5E−02	5.0E−01	2.5E−02
	1,000	2.0E−01	4.5E−02	6.0E−01	2.5E−02
	2,000	2.5E−01	4.6E−02	6.3E−01	2.8E−02
	3,000	2.8E−01	4.6E−02	6.5E−01	3.3 E−02
	5,000	3.5E−01	4.7E−02	6.8E−01	4.0E−02

*ε_r_*	10	3.0E+04	1.0E+03	3.0E+04	5.5E+02
	20	2.7E+04	6.0E+02	2.0E+04	4.8E+02
	50	2.3E+04	3.0E+02	1.2E+04	3.4E+02
	100	2.0E+04	1.0E+02	8.0E+03	2.5E+02
	200	1.4E+04	6.5E+01	6.3E+03	2.1E+02
	500	8.0E+03	4.0E+01	4.0E+03	2.0E+02
	1,000	3.5E+03	3.0E+01	3.0E+03	1.6E+02
	2,000	1.5E+03	2.6E+01	2.2E+03	1.3E+02
	3,000	8.2E+02	2.4E+01	1.2E+03	1.0E+02
	5,000	5.0E+02	2.0E+01	4.0E+02	6.0E+01
